# Supplementary material for: Physical activity habits and preferences in the month prior to a first-ever stroke
Source: PeerJ. 2014 Jul 10;2:e489. doi: 10.7717/peerj.489 (PMC4103086; doi:10.7717/peerj.489)
Supplement: Supplemental Information 1 [file peerj-02-489-s001.docx]

31/10/2011

Dear Applicant

Re: Ethics protocol "What are the barriers to maintaining or increasing physical activity levels following stroke?"

Thank you for submitting your ethics protocol for consideration. Your protocol has been considered by the E1 Committee Review Group.

I am pleased to advise that your protocol has been granted ethics approval and meets the requirements of the National Statement on Ethical Conduct in Human Research. Please note that the E1 Committee Review Group's decision will be reported to the next meeting of the Human Research Ethics Committee for endorsement.

Please regard this email as formal notification of approval.

Ethics approval is always made on the basis of a number of conditions detailed at <http://www.unisa.edu.au/res/forms/docs/humanresearchethics_conditions.doc>; it is important that you are familiar with, and abide by, these conditions. It is also essential that you conduct all research according to UniSA guidelines, which can be found at <http://www.unisa.edu.au/res/ethics/default.asp>

Please note, if your project is a clinical trial you are required to register it in a publicly accessible trials registry prior to enrolment of the first participant (e.g. Australian New Zealand Clinical Trials Registry <http://www.anzctr.org.au/Survey/UserQuestion.aspx>) as a condition of ethics approval.

Best wishes for your research.

Executive Officer

UniSA's Human Research Ethics Committee CRICOS provider number 00121B

This is an automated email and cannot be replied to. Please direct your query to [researchcompliance@unisa.edu.au](mailto:researchcompliance@unisa.edu.au).
